# Targeted optimal-path problem for electric vehicles with connected charging stations

**DOI:** 10.1371/journal.pone.0220361

**Published:** 2019-08-27

**Authors:** Fengjie Fu, Hongzhao Dong

**Affiliations:** College of Mechanical Engineering, Zhejiang University of Technology, Hangzhou, Zhejiang, China; Zhejiang University, CHINA

## Abstract

Path planning for electric vehicles (EVs) can alleviate the limited cruising range and “range anxiety”. Many existing path optimization models cannot produce satisfactory solutions due to the imposition of too many assumptions and simplifications. The targeted optimal-path problem for electric vehicles (EV-TOP), which is proposed in the paper, aims at identifying the targeted optimal path for EVs under the limited battery level. It minimizes the travel cost, which is composed of the travel time and the total time that is spent at charging stations (CSs). The model is much more realistic due to the prediction and the consideration of the waiting times at CSs and more accurate approximations of the electricity consumption function and the charging function. Charging station information and the road traffic state are utilized to calculate the travel cost. The EV-TOP is decomposed into two subproblems: a constrained optimal path problem in the network (EV1-COP) and a shortest path problem in the meta-network (EV2-SP). To solve the EV1-COP, the Lagrangian relaxation algorithm, the simple efficient approximation (SEA) algorithm, and the Martins (MS) deletion algorithm are used. The EV2-SP is solved using Dijkstra’s algorithm. Thus, a polynomial-time approximation algorithm for the EV-TOP is developed. Finally, two computational studies are presented. The first study assesses the performance of the travel cost method. The second study evaluates the performance of our EV-TOP by comparing it with a well-known method.

## Introduction

In the last few years, governments and automotive companies have recognized the potential contributions of electric vehicles (EVs) to energy saving and environmental protection [1,2]. They have been encouraging the ownership of EVs through economic incentives, which include purchase subsidies, tax exemptions, tax credits, and electricity subsidies [3–5]. However, compared with gasoline vehicles, electric vehicles face limited cruising ranges, long charging times, and remote charging stations [6]. EV drivers may prefer gasoline vehicles due to their “range anxiety” [7]. Therefore, the development of battery technology, the construction and layout of charging facilities, and path planning have become essential for overcoming the above problems. Currently, many automakers and battery manufacturers are developing new technologies for improving EV autonomy and reducing charging time. For example, General Motors reinvested USD 20 million into the GM Global Battery Systems Lab for the development of new battery technologies [8]. However, it takes too long to complete the technology transfer from research to commercialization. In addition, cities around the world are installing charging stations for EVs in busy areas [9]. The construction of charging facilities is also vigorously promoted throughout China. According to “The 13th Five-Year Plan for Energy Development”, by 2020, more than 800 new quick charging stations, 12000 centralized charging/exchanging stations, and 4.8 million decentralized charging piles will have been built for EVs. Finally, the “Four Vertical and Four Horizontal” intercity quick charging network will be constructed. It aims at meeting the charging demand of 5 million electric vehicles nationwide. This objective cannot be realized unless these charging stations are distributed reasonably [10]. Therefore, many research teams are focusing on the layout planning of charging stations [11–13]. However, subject to land use, grid load, and urban infrastructure restrictions, it is highly difficult to implement the optimal layout. Consequently, many drivers may remain unable to arrive at the nearest charging station prior to running out of battery. Others may need to join a queue at the CSs that they selected, while there are unoccupied piles at other stations. “Range anxiety” still troubles the drivers of EVs [14].

Path planning for EVs, which includes optimal routing and charging planning, can provide drivers the least-cost path and the CS/moment at which to charge, rather than just the shortest path or the nearest station. Drivers can charge at an unoccupied charging pile timely along the way and arrive at the destination while incurring the minimal cost (in terms of time, energy or money). Therefore, this paper focuses on the problem of path planning with the objective of relieving “range anxiety”.

## Literature review

### Optimal path methods for EVs

The problem of routing a vehicle with limited fuel and refueling was originally defined by Ichimori et al. [15]. The vehicle starts at a specified origin, stops and refuels at refueling vertices on the way and arrives at a specified destination. Its algorithm is a two-stage shortest path computation: constructing a new network that is composed of all the shortest paths between all pairs of refueling vertices (shorter than the cruising range) and finding the shortest path between the origin and the destination in the new network. The well-known Dijkstra’s algorithm [16] is applied to solve the problem. Lawler considered the same problem but regarded the travel time instead of the length as the cost of the arc [17]. He developed two polynomial algorithms by applying the Bellman-Ford method and the Floyd-Warshall method [18,19]. However, both objective functions neglect the impact of refueling operations. This may result in vehicles refueling more often than necessary or refueling even when the energy level is high. To limit the number of stops for refueling, the two-stage shortest path problem (SP) of EVs was modified by Alder et al. [20]. Two scenarios (the maximum number of stops for refueling was specified versus not specified) are studied to identify a more reasonable path in the electric vehicle shortest walk problem (EV-SWP). The paper also provides polynomial algorithms. The EV-SWP has been frequently reviewed and compared by other studies. However, the maximum number of stops for charging instead of the impact of refueling operations is considered. The value of the maximum number is difficult to determine and may affect the solution.

The optimal routing problem with limited fuel is equivalent to the weight-constrained shortest path problem (WCSP) if the weight (fuel consumption) and the cost of each arc are not related and vehicles cannot refuel [21,22]. Therefore, modified WCSPs were presented in other studies. Smith et al. [23] reviewed the WCSP with refueling and presented new algorithms that exploit the inter-replenishment path structure. The WCSP is NP-hard and has been discussed extensively in the literature [24–26]. Thus, Smith et al. focused on the processing method, the algorithm presentation, and the result comparison instead of the problem definition. The cost of the optimal path in the studies that are discussed above is the travel time or length. According to Artmeier et al. [27], the objective of the routing problem of EVs is to find energy-efficient paths rather than short or fast paths due to the limited battery capacity. They formalized the energy-optimal routing problem as a special case of the WCSP: the energy value may be negative, which corresponds to energy recuperation. A prefix-bounded shortest path tree with respect to function absorp is computed to solve the problem. The energy consumptions are constant among arcs and are unrelated to the arc length and the traffic state. However, in practice, EVs may become more fuel efficient as the average speed increases, particularly at local arterials [28]. To the best of our knowledge, only Goeke and Schneider and Lin et al. computed consumptions over actual road networks while considering the parameters and loads of the EV [29,30]. In this paper, we assume that the energy (electricity) consumption of an EV that is traversing a link is related to the distance and to the average travel speed. Many methods are available for estimating the average travel time and travel speed [31,32] using automatic number plate recognition data. Many other studies about urban road transportation, which include queue length estimation, traffic demand estimation, and saturation degree estimation, are also based on such information [33–35]. Consequently, ANPR data are utilized in this paper for average travel time and travel speed estimation.

### Charging planning

None of these optimal routing problems of EVs consider charging planning according to the battery charging process, the station capacities, and the levels of service (LOSs) at the charging stations (CSs).

In terms of the battery charging process, the research on the electric vehicle routing problem (EV-RP) provides detailed guidance on the charging policy and the charging function approximation. The charging policies can be classified into two groups according to the assumptions: full charging policies [36] and partial charging policies [37]. Under a full charging policy, the battery capacity is fully restored every time an EV reaches a CS, while under a partial charging policy, the level of charge (or, equivalently, the time that is spent at each CS) is a decision variable [38]. By analyzing the data from quick charging piles at various public CSs (the vehicle ID, the charging time, the charging electricity, the levels of battery at the beginning and the end, and the charging price), we find that most EVs are fully charged (approximately 85% of the battery), while a few EVs during commuting hours are not. This may be because the drivers of the EVs in the latter case are anxious to reach their destinations and there are CSs at the destinations. They prefer partial charging on the way and recharging again at the destinations. Therefore, we study an optimal routing problem under these two types of charging policies. In addition, the charging function approximations are divided into three groups: constant charging times [6,39], linear charging functions [36,40] and nonlinear charging functions [38]. In practice, the charging time that is spent at each CS depends on the charging rate and the battery level when the EV arrives/leaves. Furthermore, the charging process is divided into two phases at a specified maximum value of the battery’s terminal voltage: In the first phase, the battery level increases quickly and linearly with time. In the second phase, the battery level increases slowly and concavely with time [38]. Therefore, a constant time is not reasonable unless a depleted battery is replaced with a fully charged one. A linear charging function cannot accurately calculate the charging time similar to a nonlinear function unless EVs partially charge at CSs. Thus, a piecewise charging function is used in this paper.

In terms of the station capacities and the LOSs at the charging stations, most research on EV-RP assumes that the CSs can simultaneously handle an unlimited number of EVs. In practice, each CS is typically equipped with only a few chargers. EVs may join a queue for charging. Their travel costs will increase substantially due to the waiting time. In the optimal routing problem that is proposed here, the predicted waiting time is an important component of the travel cost. The CS capacity and the level of service (LOS) play restrictive roles. Few studies have been conducted on the estimation and prediction of the waiting time at the CS. Hanabusa and Horiguchi formulate the waiting time according to M/M/1 queueing theory from the relationship between the electric capacity of the CS and the electric demand of EVs for charging [41]. Keskin et al. handle time-dependent waiting times by discretizing the time and dividing the planning horizon into a set of time intervals. The arrival rate of EVs is approximated as a piecewise linear function of time. For an EV that is arriving at any CS in any time interval, the expected waiting time in the queue can be calculated using the arrival rate approximation and the steady-state equations of the M/G/1 queueing system [42]. Both methods assume that each charging station is equipped with a single charger and calculate the arrival rate using the historical data. Furthermore, they provide only an average waiting time for all the EVs during the same interval. However, there is often more than one charger at a single CS. The waiting time also varies with the arrivals, preferences, and charging times of EVs, the CSs, and the time periods. Thus, reliance on an average estimate of the waiting time may result in a large error. A distribution can provide both the waiting time and its reliability. Given a waiting time distribution at each CS during each time period, EVs that vary in terms of reliability preferences can more effectively select a reasonable CS and a targeted path. Data from connected charging piles, which include historic data (vehicle ID, charging time, charging electricity, the levels of battery at the beginning and the end, charging price, etc.) and real-time data (occupancy status, vehicle ID, starting time, level of the battery, time required, etc.), provide important information for the prediction of waiting time at CSs.

The remainder of this paper is organized as follows: Section 2 formally introduces the targeted optimal-path problem for EVs (EV-TOP) by describing the problem and modeling important variables, including the prediction of waiting times at CSs, the selected charging stations, the congestion-dependent electricity consumption, and the piecewise charging function. Section 3 develops a polynomial-time approximation algorithm by solving the two subproblems. Section 4 evaluates the EV-TOP by describing the study area and presenting two computational experiments. Finally, Section 5 presents the conclusions of the paper and discusses future research.

## Targeted optimal-path problem for electric vehicles

The prototypical problem that we study is to identify the targeted optimal path for an EV from its origin to its destination. The EV may charge once or more times at CSs. Consequently, any solution path is composed of several segments, namely, subpaths. All the subpaths are limited by the battery level. The optimal path refers to the path that corresponds to the lowest time cost for both basic travel and charging operations. The targeted optimal path is identified via the proposed travel-cost method.

### Problem description

We consider a regional road network, which is represented by a complete graph *G* = (*V*, *E*). The vertex set *V* = {1, 2, …, *N*} is a combination of nodes, which represent conventional intersections (CIs) and charging stations (CSs); |*V*| = *N*. The arc set *E* = {(*i*, *j*), ∀*i*, *j*ϵ *V*} corresponds to the links that connect two neighboring nodes in *V*; |*E*| = *M*. Each link is associated with four nonnegative attributes: travel time *t*_*ij*_, travel speed *v*_*ij*_, electricity consumption *e*_*ij*_, and distance *l*_*ij*_. *t*_*ij*_ is the average travel time of all the vehicles on the same link from the collected data (e.g., automatic number plate recognition data). It is updated every time interval *T* (e.g., 5 min) in real time. Travel speeds are assumed to be constant over each link.

The origin and destination nodes of an EV are denoted by *o* and *d*, where *o*, *d*ϵ *V*. The EV has an initial battery level of *B*_0_, which ranges from *B*_min_ to *B* at *o*, where *B* is the battery capacity and *B*_min_ is the minimum reserve level. All the CSs are public quick charging stations. They vary in terms of capacity. The impact of charging operations is considered: the travel cost of the EV is composed of the travel time and the total time that is spent at CSs (a sum of the waiting time *w*, the base time loss *f*, and the charging time *c*). Consequently, as the number of stops for charging *p* increases, the travel cost may increase. Therefore, here, no limit is imposed on the number of stops *p*.

Binary variables *x*_*ijk*_ and *y*_*jk*_ are defined to denote the selection of links and CSs. *x*_*ijk*_ is equal to 1 if the EV traverses link (*i*,*j*)ϵ*E* over subpath *k =* 1, 2, …, *p*+1 and to 0 otherwise. *y*_*jk*_ is equal to 1 if the EV charges at CS *j* over subpath *k* and to 0 otherwise.

Finally, we define the EV-TOP as the problem of finding the least-cost path that begins at *o*, charges at CSs in *S*, and ends at *d*. Feasible solutions to the EV-TOP satisfy the following conditions: 1) the travel cost is minimal; 2) the electricity consumption over any subpath that begins at *o* and ends at a node in *d*∪ *S* is less than *B*_0_*-B*_min_; and 3) the electricity consumption over any subpath that starts at a node in *S* and ends at a node in *d*∪ *S* is less than *B*’*- B*_min_, where *B*’ is the battery level when the EV finishes charging and is also the initial battery level over the next subpath. The integer programming formulation of the EV-TOP, which uses decision variables *x*_*ijk*_ and *y*_*jk*_, is as follows:

$$\underset{X}{\min}\sum_{k=1}^{p+1}\left[\sum_{i,j:\left(i,j\right)\in E}t_{ij}x_{ijk}+\sum_{j\in S'}\left(f_{j}+w_{j}+c_{jk}\right)y_{jk}\right]$$ (1)

$$\mathrm{subject}\;\;\mathrm{to}:\;f_{1}\left(x_{ijk}\right)-f_{2}\left(x_{jik}\right)=0$$ (2)

$$\sum_{j\in S'}y_{jk}=1,\forall j\in S',k\leq p$$ (3)

$$\sum_{j\in S'}\sum_{i:\left(i,j\right)\in E}x_{ijk}y_{jk}=1,\forall j\in S',\left(i,j\right)\in E,k\leq p$$ (4)

$$\sum_{i,j:\left(i,j\right)\in E}e_{ij}x_{ijk}\leq B{'}_{k}-B_{\min},\;k=2,3,\ldots ,p+1$$ (5)

$$\sum_{i,j:\left(i,j\right)\in E}e_{ij}x_{ij1}\leq B_{0}-B_{\min}$$ (6)

$$x_{ijk}=\left\{0,1\right\},\;\forall i,j\in V:\left(i,j\right)\in E,k=1,2,\ldots ,p+1$$ (7)

$$y_{jk}=\left\{0,1\right\},\;\forall j\in S',k=1,2,\ldots ,p$$ (8)

Eq (1) is the objective function, which minimizes the travel cost. Via the consideration of charging operations, the generation of solutions with excessive charging stops can be avoided. A vector ***X*** denotes a path between *o* and *d*. It is a sequence of vertices. The base time loss *f*_*j*_ is the minimum amount of time that is spent at CS *j*, which includes driving into the station, looking for an unoccupied charging pile, paying, and leaving the station. As the name indicates, it is a constant and it depends on the location, the scale, and the parking rules of each CS. Its value can be determined via investigation. *w*_*j*_ and *c*_*jk*_ denote the waiting time and the charging time, respectively, at CS *j* over subpath *k*. A prediction method for the waiting time at a CS and a piecewise linear charging function with 2 pieces are proposed in Section *Modeling of variables*. They are used to calculate *w*_*j*_ and *c*_*jk*_. *S*’ denotes the available CS set for the EV, where *S*’ ϵ*S*. It depends on the location, the typical paths, and the waiting time reliability of the EV and is described in detail in Section *Modeling of variables*.

Constraint (2) ensures that the conservation laws hold for each vertex: the number of outgoing arcs equals the number of incoming arcs at each vertex. The functions *f*_1_ and *f*_2_ depend on the types of the vertices and the sequence number of the subpath; additional details will be provided in a later section. According to Constraints (3) and (4), the EV must charge at one (at most) of the CSs in *S*’ and the EV cannot charge at CS *j* unless it passes link (*i*,*j*). Constraints (5) and (6) limit the electricity consumption over the subpaths to ensure that EVs can arrive at the ending vertex prior to running out of battery. The limited electricity consumption depends on the initial battery levels (*B*_0_ and *B*’) at the beginning vertex over the first subpath or other subpaths. It will be calculated in Section *Modeling of variables*. The decision variables are expressed in Constraints (7) and (8).

### Modeling of variables

#### Prediction of the waiting times at CSs

We assume that the arrival of EVs obeys a Poisson distribution with parameter *λt* and the charging time obeys an exponential distribution with parameter 1/*μ*. The hypothesis is supported by the one-sample Kolmogorov-Smirnov (K-S) test. The test results that are obtained using historical data (the arrivals and the charging times of EVs) for 10 CSs during every 60-min interval are presented in Table 1. Therefore, the prediction of the waiting time distribution can be based on an M/M/n/m/∞/C queueing model. The probability that an EV must wait longer than *t* at a CS is calculated via Eqs (9) and (10).

**Table 1:** One-sample Kolmogorov-Smirnov test results of arrivals and charging times.

Data group	Arrivals			Charging time		
	Sample size	Mean value (vehicles/minute)	*p*-value	Sample size	Mean value (minute)	*p*-value
**1**	30	0.93	0.59	56	22.32	0.57
**2**	30	0.16	0.42	9	23.56	0.66
**3**	30	0.10	0.48	6	20.58	0.42
**4**	30	0.47	0.54	28	25.27	0.63
**5**	30	0.08	0.76	5	21.18	0.72
**6**	30	0.02	0.64	11	26.25	0.55
**7**	30	0.43	0.69	26	27.31	0.62
**8**	30	0.99	0.57	59	21.63	0.52
**9**	30	0.08	0.53	5	30.27	0.65
**10**	30	0.18	0.61	11	26.61	0.48
**11**	30	0.39	0.62	24	32.54	0.47
**12**	30	0.18	0.49	11	24.22	0.54

$$P\{w_{j}\leq t\}=1-P\{w_{j}>0\}\exp\left[-\left(n_{j}-R_{j}\right)\mu_{j}t\right],t\geq 0,R_{j}=\lambda_{j}/\mu_{j}<n_{j}\leq m_{j}$$ (9)

$$P\{w_{j}>0\}=\frac{\frac{R_{j}^{n}}{n_{j}!}\left(\frac{n_{j}}{n_{j}-R_{j}}\right)}{\sum_{i=0}^{n-1}\frac{R_{j}^{i}}{i!}+\frac{R_{j}^{n}}{n_{j}!}\left(\frac{n_{j}}{n_{j}-R_{j}}\right)}$$ (10)

where *λ*_*j*_ is the average arrival rate of EVs at CS *j*; *μ*_*j*_ is the average service rate of CS *j*; *n*_*j*_, *m*_*j*_ and *R*_*j*_ are the number of chargers, the parking capacity, and the offered load, respectively, at CS *j*; and *R*_*j*_ <*n*_*j*_ *≤m*_*j*_ is satisfied to ensure the stability of the M/M/n/m/∞/C queueing system.

The average arrival rate varies substantially among the time periods, while the average charging time is stable. Therefore, a prediction of *λ*_*j*_ instead of a historical value can improve the prediction of the waiting time distribution. Given the number of EVs that must be charged during time period (*t*_0_,*t*_0_+∆*t*) in the network and the fraction of the EVs that select CS *j*, the prediction of the average arrival rate at CS *j* is expressed as Eq (11).

$$\lambda_{j}=Q_{c}P_{j}/\Delta t=Q_{c}\sum_{i=1}^{Q_{c}}P_{ij}/\Delta t$$ (11)

where *Q*_c_ denotes the number of EVs that must be charged during time period (*t*_0_,*t*_0_+∆*t*) in the network, *P*_*j*_ denotes the fraction of EVs that select CS *j*, and *P*_*ij*_ denotes the probability that EV *i* chooses CS *j* for charging. The value of *Q*_c_ is known with connected charging stations. *P*_*ij*_ is modeled using the multinomial logit model as follows:

$$\left\{ \begin{array}{l} U_{ij}=\varphi_{1}z_{ij,1}+\varphi_{2}z_{ij,2}+\varepsilon_{ij}\\ \varepsilon_{ij}∼\mathrm{the}\;\mathrm{extreme}\;\mathrm{value}\;\mathrm{distribution} \end{array}\right.$$ (12)

$$P_{ij}=\exp\left(\varphi_{1}z_{ij,1}+\varphi_{2}z_{ij,2}\right)/\sum_{j=1}^{J}\exp\left(\varphi_{1}z_{ij,1}+\varphi_{2}z_{ij,2}\right)$$ (13)

where *U*_*ij*_ is the utility function for EV *i* selecting CS *j* for charging; *z*_*ij*,1_ and *z*_*ij*,2_ denote variables that are related to CS *j* for EV *i* and depend on the estimated average waiting time at the CS and the distance to the CS, respectively; *ɛ*_*ij*_ captures the collective impact of all unobserved factors that affect the EV’s choice; and *φ*_1_ and *φ*_2_ are the coefficients of *z*_*ij*,1_ and *z*_*ij*,2_, respectively. EVs differ in terms of waiting time reliability preferences due to differences in the travel objective, the driving habits, and the battery level, among other factors. Therefore, the predicted waiting time depends on the probability threshold, as expressed by Eq (14).

$$w_{j}=w|P\{w_{j}\leq w\}=\alpha$$ (14)

where *w*_*j*_ denotes the predicted waiting time at CS *j*; *α* is the probability threshold, which corresponds to the reliability preference of the EV; and *w* is a value that satisfies *P*{*w*_*j*_≤*w*} = *a*.

#### Description of the selected charging station

An EV always charges at CSs along feasible paths. Therefore, removing CSs that are far away from the most frequent paths from the set of possible solutions will improve the algorithm. The available CS set *S*’ satisfies Eq (15).

$$\{S'=\left\{s:\forall s\in S,l_{s,z}\leq l^{\max},z=1,2,\ldots ,Z\right\},l_{s,z}=\min{\left\{l_{s,z,i}\right\}}_{i:\left(i.j\right)\in P^{z}},P^{z}={\{\ldots \to \left(i,j\right)\to \ldots \}}_{i,j:\left(i.j\right)\in E}$$ (15)

A charging station *s* in the available CS set *S*’ must have a distance *l*_*s*,*z*_ to any typical path *P*^*z*^ that does not exceed a threshold value *l*^max^. *l*_*s*,*z*_ is the minimum of the Euclidean distances from *s* to all the nodes along the typical path *P*^*z*^. *l*_*s*,*z*,*i*_ denotes the Euclidean distance from *s* to node *i* along *P*^*z*^. The typical paths between a pair of nodes correspond to the most frequent *Z* paths that are identified using historical data (GPS data or ANPR data).

#### Congestion-dependent electricity consumption

As discussed above, the electricity consumption *e*_*ij*_ over a link is related to the distance *l*_*ij*_ and the travel speed *v*_*ij*_. The EV will consume more electricity when it drives too slow or too fast. The value of *e*_*ij*_ is calculated via Eq (16).

$$e_{ij}=\psi \times l_{ij}\times \left(1+\theta \times \left|v_{ij}-v^{b}\right|/v^{b}\right)$$ (16)

where *ψ* denotes the base energy consumption rate, namely, the electricity consumption per kilometer at the optimal speed *v*^b^ (which is equal to 45 km/h in the paper) and *θ* is the influence factor of the difference between the actual speed and the optimal speed. Thus, the battery level *b*_*jk*_ of the EV that arrives at CS *j* over subpath *k* is as follows:

$$b_{jk}=\left\{ \begin{array}{l} B{'}_{k}-\sum_{i,j:\left(i,j\right)\in E}e_{ij}x_{ijk},1<k\leq p\\ B_{0}-\sum_{i,j:\left(i,j\right)\in E}e_{ij}x_{ij1} \end{array}\right.$$ (17)

where *B*’_*k*_ is the initial battery level over subpath *k* and the battery level when the EV finishes charging over subpath *k-*1. Its value depends on the charging policies and the subpath.

#### Piecewise charging function

**Fig 1:**
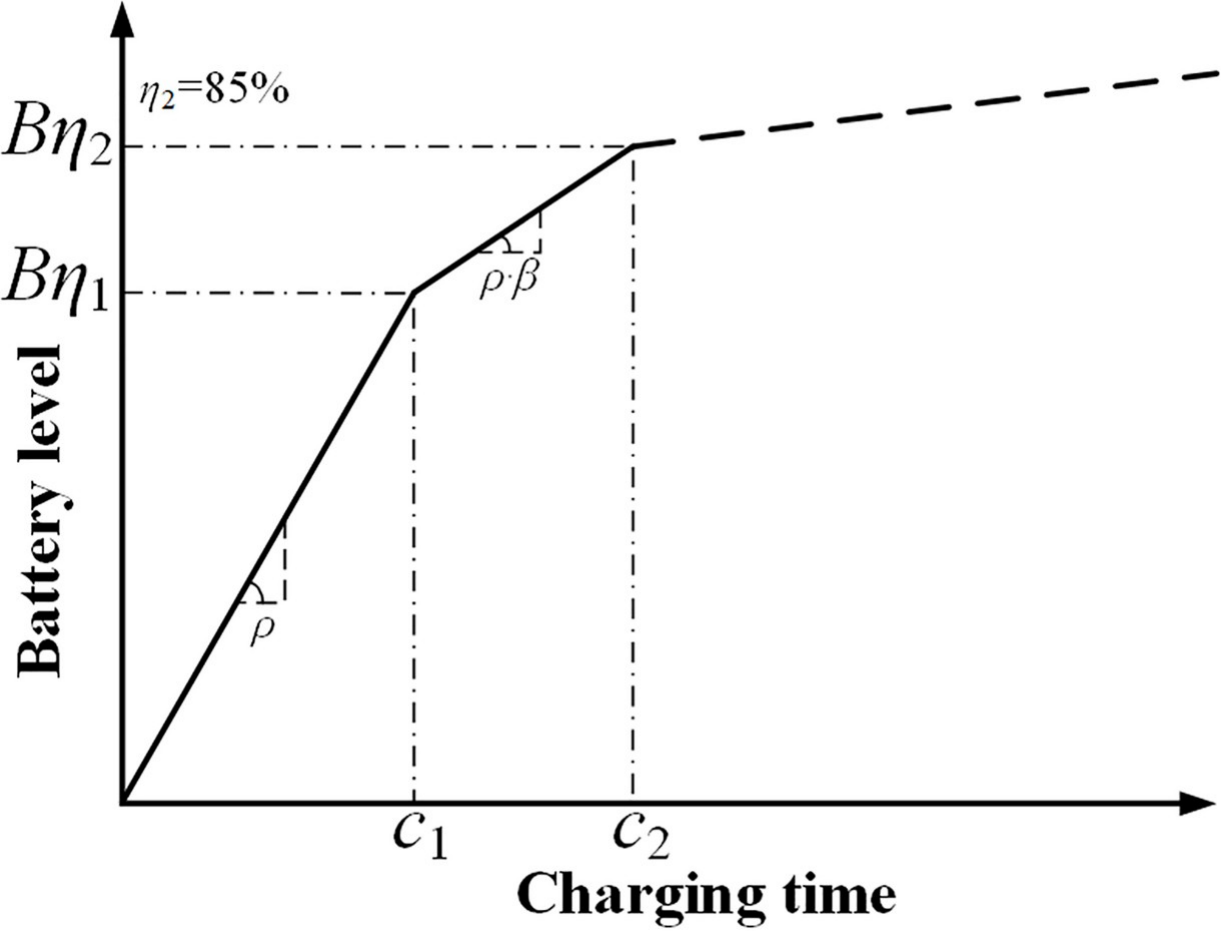
Piecewise linear charging function with 2 pieces.

The charging process at a charging pile of *ρ* kw is approximated using a piecewise linear function with 2 pieces, as presented in Fig 1. In the first stage, the actual charging rate is *ρ* and the maximum battery level is *Bη*_1_. In the second stage, the actual charging rate is *ρ∙β* and the maximum battery level is *Bη*_2_. *β* denotes the attenuation rate and *β<*1. As discussed in Section *Literature*, most EVs that are fully charging at public quick CSs typically sacrifice the last 15% capacity of the battery to save time and money, namely, *η*_2_ = 85%. If the EV arrives at CS *j* over subpath *k* with a battery level of *b*_*jk*_, its charging time *c*_*jk*_ under various charging policies that are based on the piecewise linear function is calculated via Eq (18).

$$c_{jk}=\left\{ \begin{array}{l} \frac{B{'}_{k+1}-B\eta_{1}}{\rho \beta }+\frac{B\eta_{1}-b_{jk}}{\rho },b_{jk}>B\eta_{1},k\leq p\\ \frac{B{'}_{k+1}-b_{jk}}{\rho \beta }+\frac{B\eta_{1}}{\rho },\;b_{jk}\leq B\eta_{1},k\leq p \end{array}\right.$$ (18)

The EV will not prefer partial charging unless it’s over subpath *p*. Thus, *B*’_*k*_ will be equal to *Bη*_2_ if *k*≤*p*, as expressed in Eq (19). Over the *p*th subpath, EVs may prefer to utilize the partial charging policy to reduce the total travel time. They will stop charging once the battery level *B*’_*p*+1_ reaches *Bη*_2_*γ*, as expressed in Eq (20), in which *γ* denotes the partial rate and *γ* <1. Its value depends on the EV’s preferences.

$$B{'}_{k}=B\eta_{2},\;1<k\leq p$$ (19)

$$B{'}_{p+1}=\left\{ \begin{array}{l} B\eta_{2}\gamma ,\delta =1,B\eta_{2}\gamma >B{'}_{p}\\ B\eta_{2},\;\delta =0 \end{array}\right.$$ (20)

where *δ* is a charging policy indicator and *δ =* 0 or 1 indicates whether the EV chooses partial charging over the *p*th subpath or not.

### Solving the EV-TOP

#### Two-stage shortest path problem

**Fig 2:**
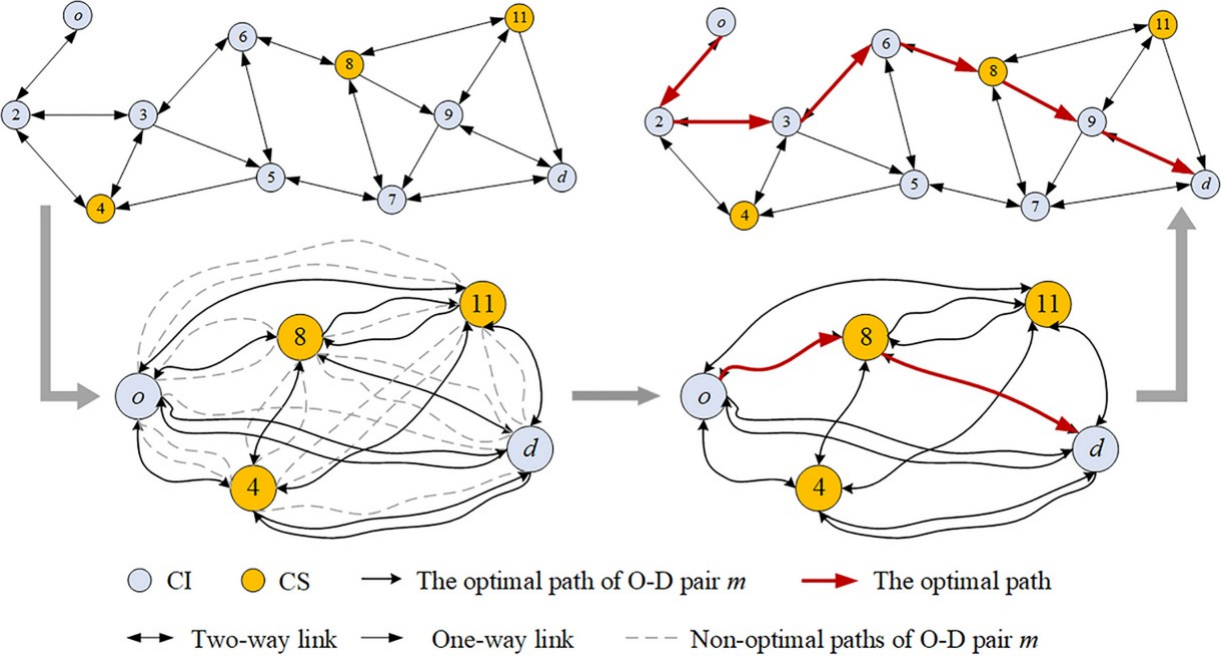
Process of the transformation from the EV-TOP to the EV1-COP and the EV2-SP.

The CSP and the WCSP are known to be NP-hard. Since they are special cases of the EV-TOP, the EV-TOP is also NP-hard. Thus, the EV-TOP is transformed into a two-stage shortest path problem. In the first stage, the constrained shortest path problem in *G* = (*V*, *E*), namely, the EV1-COP for short, aims at identifying all the optimal paths between any two vertices among the origin, the destination, and *S*’. It is a special case of the EV-TOP in which *p* = 0. Consequently, a meta-network *G*’ = (*V*’, *E*’) is constructed. The meta-node set *V*’ is composed of the originating and destination vertices and the available CS set *S*’, where |*V*’| = *N*’ = |*S*’|+2. All the optimal paths between each O-D pair compose the meta-arc set *E*’ = {(*o*^*m*^, *d*^*m*^), ∀*o*^*m*^, *d*^*m*^∈ *V*’, *m* = 1, …, *M*’}, where |*E*’| = *M*’. (*o*^*m*^, *d*^*m*^) denotes the optimal path or an O-D pair. In the second stage, the non-constrained shortest path problem, namely, the EV2-SP for short, aims at identifying the least-cost path between the origin *o* and the destination *d* in *G*’ = (*V*’, *E*’). Fig 2 illustrates the process.

A similar formulation of the EV1-COP_*m*_ for any O-D pair (*o*^*m*^, *d*^*m*^) is as follows:

$$f\left(x_{m}\right)=\underset{x_{m}}{\min}\sum_{i,j:\left(i,j\right)\in E}t_{ij}x_{ij}^{m}$$ (21)

such that

$$\sum_{j:\left(i,j\right)\in E}x_{ij}^{m}-\sum_{j:\left(j,i\right)\in E}x_{ji}^{m}=\left\{ \begin{array}{l} 1,i=o^{m},\forall i\in V\\ 0,i\notin \left\{o^{m},d^{m}\right\},\forall i\in V\\ -1,i=d^{m},\forall i\in V \end{array}\right.$$ (22)

$$\sum_{i,j:\left(i,j\right)\in E}e_{ij}x_{ij}^{m}\leq \left\{ \begin{array}{l} B\eta_{2}\gamma -B_{\min},o^{m}\neq o,d^{m}=d\;\mathrm{and}\;\delta =1\\ B\eta_{2}-B_{\min},o^{m}\neq o,d^{m}\neq d\;\mathrm{or}\;\delta =0\\ B_{0}-B_{\min},\;o^{m}=o \end{array}\right.$$ (23)

$$x_{ij}^{m}=\left\{0,1\right\},\;\forall i,j\in V:\left(i,j\right)\in E$$ (24)

The objective of the EV1-COP_*m*_ is to minimize the travel time (denoted by *f*(***x***_*m*_)) over the path between O-D pair *m*, as expressed in Eq (21). A vector ***x***_*m*_ denotes a path between O-D pair *m*. It is a sequence of vertices. Constraint (22) ensures that the conservation laws are obeyed and the origin and destination are as specified: 1) the number of incoming arcs at vertex *o*^*m*^ must be one less than the number of outgoing arcs at vertex *o*^*m*^ to ensure that *o*^*m*^ is the origin; 2) the number of incoming arcs must be equal to the number of outgoing arcs at any vertex *i* to ensure the conservation laws if *i* is neither *o*^*m*^ nor *d*^*m*^; and 3) the number of incoming arcs at vertex *d*^*m*^ must be one more than the number of outgoing arcs at vertex *d*^*m*^ to ensure that *d*^*m*^ is the destination. Constraint (23) limits the electricity consumption over the subpaths to ensure that EVs can arrive at *d*^*m*^ prior to running out of battery. Constraint (24) defines the decision variable *x*^*m*^_*ij*_: *x*^*m*^_*ij*_ is equal to 1 if the EV traverses the link (*i*,*j*)∈ *E* between O-D pair *m =* 1, 2, …, *M*’ and to 0 otherwise.

Then, EV2-SP is formulated in Eqs (25)–(32).

$$\underset{X}{\min}\sum_{i,j\in V':\left(i,j\right)\in E'}\left[tt_{ij}X_{ij}+\left(f_{j}+w_{j}+c_{j}\right)y_{j}\right]-\delta \Delta c$$ (25)

such that

$$tt_{ij}=f\left(x_{m}^{*}\right),\;\forall i,j\in V':\left(i,j\right)\in E',i=o^{m},j=d^{m}$$ (26)

$$\sum_{j:\left(i,j\right)\in E'}X_{ij}-\sum_{j:\left(j,i\right)\in E'}X_{ji}=\left\{ \begin{array}{l} 1,i=o,\forall i\in V'\\ 0,i\notin \left\{o,d\right\},\forall i\in V'\\ -1,i=d,\forall i\in V' \end{array}\right.$$ (27)

$$w_{j}=w|P\{w_{j}\leq w\}=\alpha ,\forall j\in V',j\neq d$$ (28)

$$c_{j}=\left\{ \begin{array}{l} \frac{B\eta_{2}-B\eta_{1}}{\rho \beta }+\frac{B\eta_{1}-b_{j}}{\rho },b_{j}>B\eta_{1},\forall j\in V',j\neq d\\ \frac{B\eta_{2}-b_{j}}{\rho \beta }+\frac{B\eta_{1}}{\rho },\;b_{j}\leq B\eta_{1},\forall j\in V',j\neq d \end{array}\right.$$ (29)

$$\Delta c=\left\{ \begin{array}{l} \frac{B\eta_{2}\left(1-\gamma \right)}{\left(\rho \beta \right)},\;B\eta_{2}\gamma >B\eta_{1}\\ \frac{B\eta_{2}-B\eta_{1}}{\rho \beta }+\frac{B\eta_{1}-B\eta_{2}\gamma }{\rho },B\eta_{2}\gamma \leq B\eta_{1} \end{array}\right.$$ (30)

$$y_{j}=\left\{ \begin{array}{l} 0,j=d\\ 0,\sum_{i:\left(i,j\right)\in E'}X_{ij}=0,\forall i,j\in V',j\neq d\\ 1,\sum_{i:\left(i,j\right)\in E'}X_{ij}>0,\forall i,j\in V',j\neq d \end{array}\right.$$ (31)

$$X_{ij}=\left\{0,1\right\},\;\forall i,j\in V':\left(i,j\right)\in E'$$ (32)

Eq (25) is the objective function, which minimizes the travel cost over the path ***X***. The charging time at each CS is calculated based on the full charging policy, as expressed by Constraint (29). A charging time difference ∆*c* will be induced if the EV prefers partial charging over the last subpath. Therefore, an item *δ*∆*c* is added into Eq (25). Constraint (26) defines the cost of each meta-arc in *G*’ (denoted by *tt*_*ij*_) as the travel time over the optimal subpath x^*^_m_. Constraint (27) ensures that the conservation laws are obeyed and the origin and destination are as specified, similar to Constraint (22). Constraints (28) and (29) readjust the waiting time and the charging time of the EV at CS *j*. The charging time difference ∆*c* is calculated by Constraint (30). *y*_*j*_ indicates whether the EV charges (*y*_*j*_ = 1) or not (*y*_*j*_ = 0) at CS *j*, which is determined by meta-node *j* and *X*_*ij*_, as expressed in Constraint (31): 1) we don’t consider the charging operation at the destination node *d*, namely, if CS *j* is vertex *d*, we let *y*_*j*_ = 0; 2) the EV cannot charge at CS *j* unless the EV passes vertex *j*; and 3) the EV will charge at CS *j* once the EV passes vertex *j*. Constraint (32) defines the decision variables *X*_*ij*_ in *G*’. The final optimal path between *o* and *d* is denoted by X^*^.

#### Solution strategy

Similarly, as a special case of the EV-TOP, the EV1-COP is also NP-hard. First, a strongly polynomial-time approximation algorithm is proposed in this section for solving the EV1-COP. We apply the Lagrangian relaxation algorithm [43] using Lagrangian duality theory [44], the subgradient method and the Handler-Zang algorithm in the first step to obtain the initial lower and upper bounds. In the second step, the simple efficient approximation (SEA) algorithm [45] and the MS deletion algorithm [46] are used to obtain an *ɛ*-approximated subpath by closing the gap between the upper and lower bounds. Second, Dijkstra’s algorithm is applied to solve the EV2-SP. Thus, the EV-TOP is solved and the optimal path is determined.

##### Dual of the EV1-COP

Eq (33) defines a Lagrangian function by introducing a variable *λ*_*m*_ (the Lagrangian multiplier *λ*_*m*_*≥*0) and a relaxed Constraint (23) to the objective function of the EV1-COP_*m*_.

$$L\left(x_{m},\lambda_{m}\right)=f\left(x_{m}\right)+\lambda_{m}\cdot g\left(x_{m}\right)$$ (33)

Thus, the original EV1-COP_*m*_ is equivalent to a new relaxed problem without Constraint (23). Eq (34) presents the relationship, in which f^*^ is the optimal objective function value of the EV1-COP_*m*_.

$$f^{*}=f\left(x_{m}^{*}\right)=\underset{x_{m}}{\min}f\left(x_{m}\right)=\underset{x_{m}}{\min}\;\underset{\lambda_{m}\geq 0}{\max}L\left(x_{m},\lambda_{m}\right)$$ (34)

The dual formation, namely, EV1-COP-D_*m*_, is introduced for solving the relaxed problem, as expressed in Eq (35). L^*^ is the optimal objective function value of the EV1-COP-D_*m*_.

$$L^{*}=L\left(\lambda_{m}^{*}\right)=\underset{\lambda_{m}\geq 0}{\max}L\left(\lambda_{m}\right)=\underset{\lambda_{m}\geq 0}{\max}\;\underset{x_{m}}{\min}L\left(x_{m},\lambda_{m}\right)$$ (35)

L^*^ does not exceed f^*^. Thus, there may be a gap between the optimal objective function values of the EV1-COP_*m*_ and of the EV1-COP-D_*m*_. Hence, we solve the EV1-COP-D_*m*_ and obtain the upper and lower bounds on its optimal solution, which are denoted as *L*^ub^ and *L*^lb^.

#### *ε*-approximated subpath

Given an instance subpath ***x*** and a desired degree of accuracy *ε* > 0, subpath ***x*** is an *ε*-approximated subpath to the EV1-COP_*m*_ if Eq (35) is satisfied. It is determined using the dynamic programming algorithm with the lower and upper bounds, namely, *L*^ub^ and *L*^lb^, that were obtained in the first step.

$$\frac{\left|f^{*}-f\left(x\right)\right|}{f^{*}}\leq \varepsilon$$ (36)

where *f*(***x***) is the objective function value of the EV1-COP_*m*_ on subpath ***x***.

### Time complexity

In the first step of solving the EV1-COP_*m*_, the time complexity of the Lagrangian relaxation algorithm is O(*M*log^2^*M*)×O(*M*+ *N*log*N*) [43]. In the second step, the SEA algorithm has a time complexity of O(*MN*(loglog*N+*1*/ε*) [45]. The overall run time of the EV1-COP_*m*_ algorithm is the sum of the two terms. It is asymptotically dominated by O(*M*^2^log^2^*M+ MN*log^3^*M+MN/ε*). The construction of *G*’ must solve the EV1-COP_*m*_ *N*’-1 times. Thus, the time that is required for transforming *G* to *G*’ is O(*N*’(*M*^2^log^2^*M+ MN*log^3^*M+MN/ε*)). Furthermore, Dijkstra’s algorithm is used to solve the EV2-SP in *G*’ and its time complexity is O(*M*’+*N*’log*N*’) [47]. Overall, the time complexity of the algorithm for solving the EV-TOP is O(*N*’(*M*^2^log^2^*M+ MN*log^3^*M+MN/ε*)).

## Evaluation

The EV-SWP, which was proposed by Alder et al. [20], has been frequently reviewed and evaluated by other studies on optimal path methods for EVs. We compare our EV-TOP with the EV-SWP in the section to evaluate our proposed method. We present two computational experimental studies: The first study assesses the performance of the travel cost method, namely, more accurately approximating the electricity consumption function and considering the waiting time at CSs. Since the travel cost in the EV-SWP is the distance, the effects of charging operations are neglected. The second study evaluates the performance of our EV-TOP by comparing the optimal paths and LOSs at CSs that are based on the EV-TOP with those that are based on the EV-SWP.

### Network and parameters

The EV-TOP is evaluated in the road network *G* = (*V*,*E*) of a region in Hangzhou, with |*V|* = 49, |*E|* = 163, and |*S|* = 10. Then, we generated 20 random networks that reasonably resembled the real-word road network. The EV-TOP and the EV-SWP are tested on randomly generated data to further evaluate the significance of the improvement.

**Fig 3:**
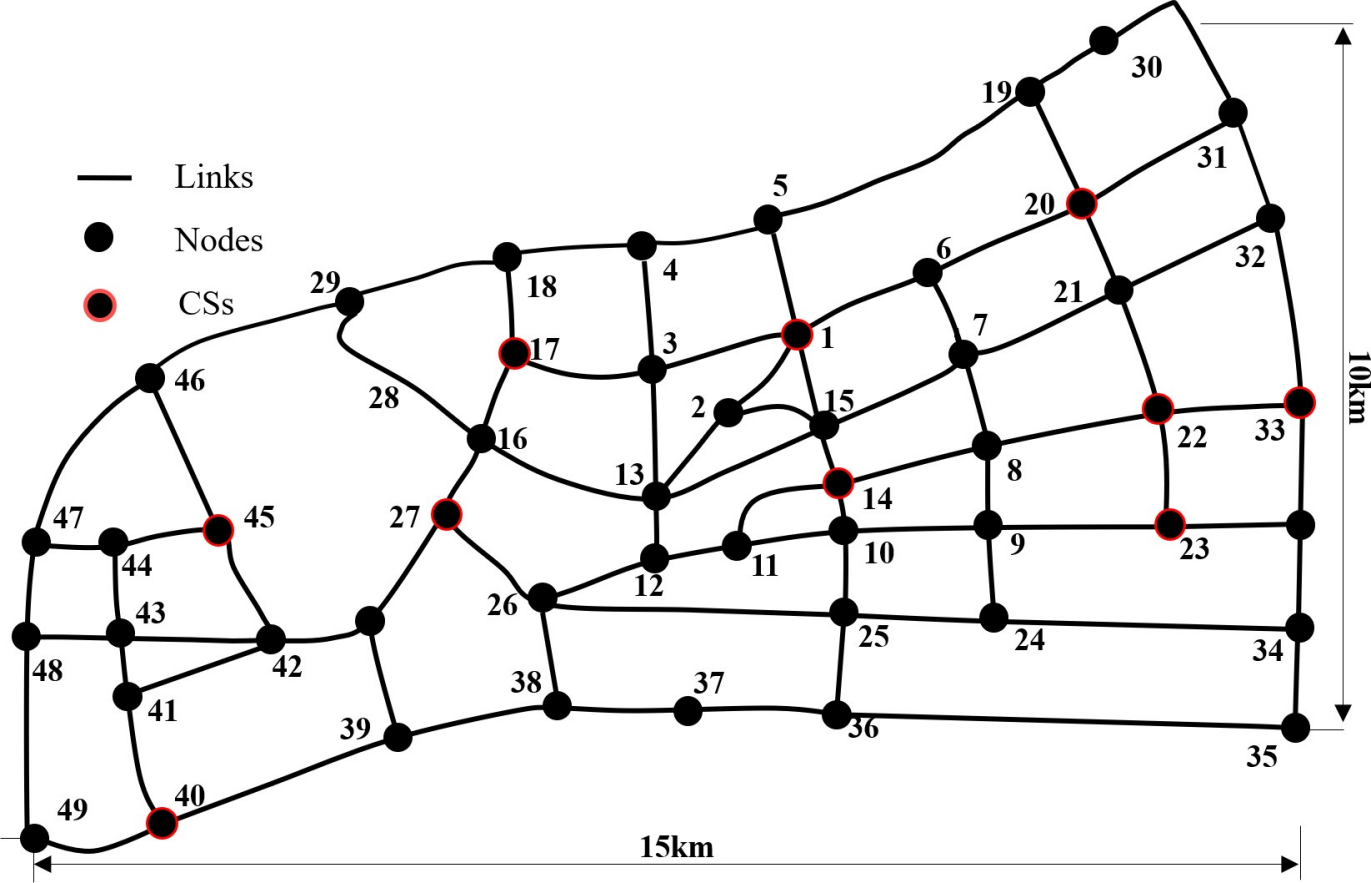
Study network for evaluation.

The study network *G* is as illustrated in Fig 3, where links are represented by black lines, nodes are represented by black points and CSs are circled in red. Its south side stretches from Node 49 in the west to Node 36 in the east, with a straight-line distance of 15 km. Its east side stretches from Node 36 in the south to Node 30 in the north with a straight-line distance of 10 km.

There are ten charging stations. Table 2 lists the number of charging piles and the base time loss at each CS.

**Table 2:** Information on the CSs.

CS	1	14	17	20	22	23	27	33	40	45
***n***_***j***_	21	4	4	12	8	6	12	24	2	8
***f***_***j***_	4.8	2.2	2.9	1.9	1.1	2.6	2.5	5.1	2	1.7

**Fig 4:**
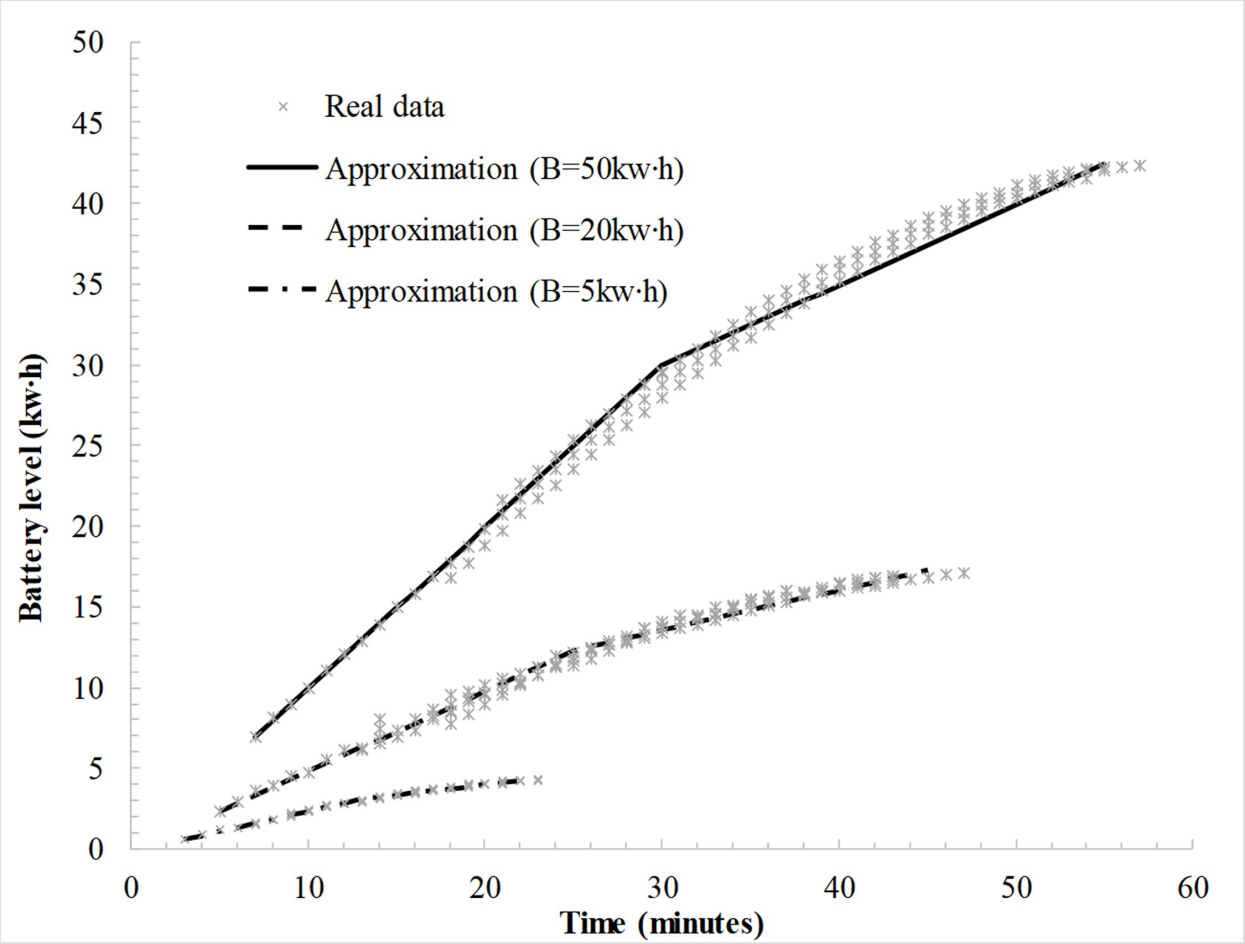
Piecewise linear approximation for three types of EVs.

For simplicity, we select three main types of EVs in the actual road network, which correspond to the development of battery technology. Their performance parameters, except *α*, which are obtained by fitting the charging function to real data, are listed in Table 3. The fitting curves are plotted in Fig 4.

**Table 3:** Performance parameters of three types of EVs.

Type	*ψ* (kw*∙*h/km)	*θ*	*β*	*η*_1_	*η*_2_	*B* (kw*∙*h)	*Ρ* (kw)
**1**	0.20	1	0.5	60	85	5	15
**2**	0.20	1	0.5	60	85	20	30
**3**	0.12	1	0.5	60	85	50	60

### Benefits of the travel cost method

#### Benefits of the electricity consumption function

The EV-CSP always limits the total distance or the total electricity consumption to avoid the EV running out of battery. However, the existing methods assume that the electricity consumption over each link is constant or related to the link length. In practice, the optimal path may become an infeasible solution. A consumption function that considers the speed over the link, as expressed in Eq (16), can better avoid this scenario. Table 4 demonstrates the advantage of using this function with an example. The base consumption rate, the initial battery level and the battery capacity of the example EV are 0.2 kw∙h/km, 3 kw∙h and 5 kw∙h, respectively, and *e*^1^/ *e*^2^ denotes the electricity consumption that is unrelated/related to the average speed over each link.

**Table 4:** Electricity consumption over each link and the least-cost path.

	1	2	3	4	5	6	7	8	9	10	11	12	13	14	15	Total
***l* (km)**	1.17	0.71	0.50	0.46	0.78	1.18	0.71	1.20	0.48	1.26	0.83	1.00	0.56	1.13	1.22	**11.43**
***v* (km/h)**	64.0	50.8	27.3	40.4	37.5	36.9	32.9	32.7	23.4	37.7	20.4	38.9	29.5	51.9	45.6	
***e***^**1**^ **(kw∙h)**	0.23	0.14	0.10	0.09	0.16	0.24	0.14	0.24	0.10	0.25	0.17	0.20	0.11	0.23	0.24	**2.63**
***e***^**2**^ **(kw∙h)**	0.33	0.16	0.14	0.10	0.18	0.28	0.18	0.31	0.14	0.29	0.26	0.23	0.15	0.26	0.25	**3.26**

According to Table 4, the total electricity consumption over the least-cost path that uses *e*^1^ is 2.63 kw∙h, which is less than 3 kw∙h. Thus, the least-cost path is exactly the optimal path. However, that value if *e*^2^ is used is 3.26 kw∙h. Hence, the least-cost path is not a feasible solution.

#### Benefits of waiting time prediction

**Fig 5:**
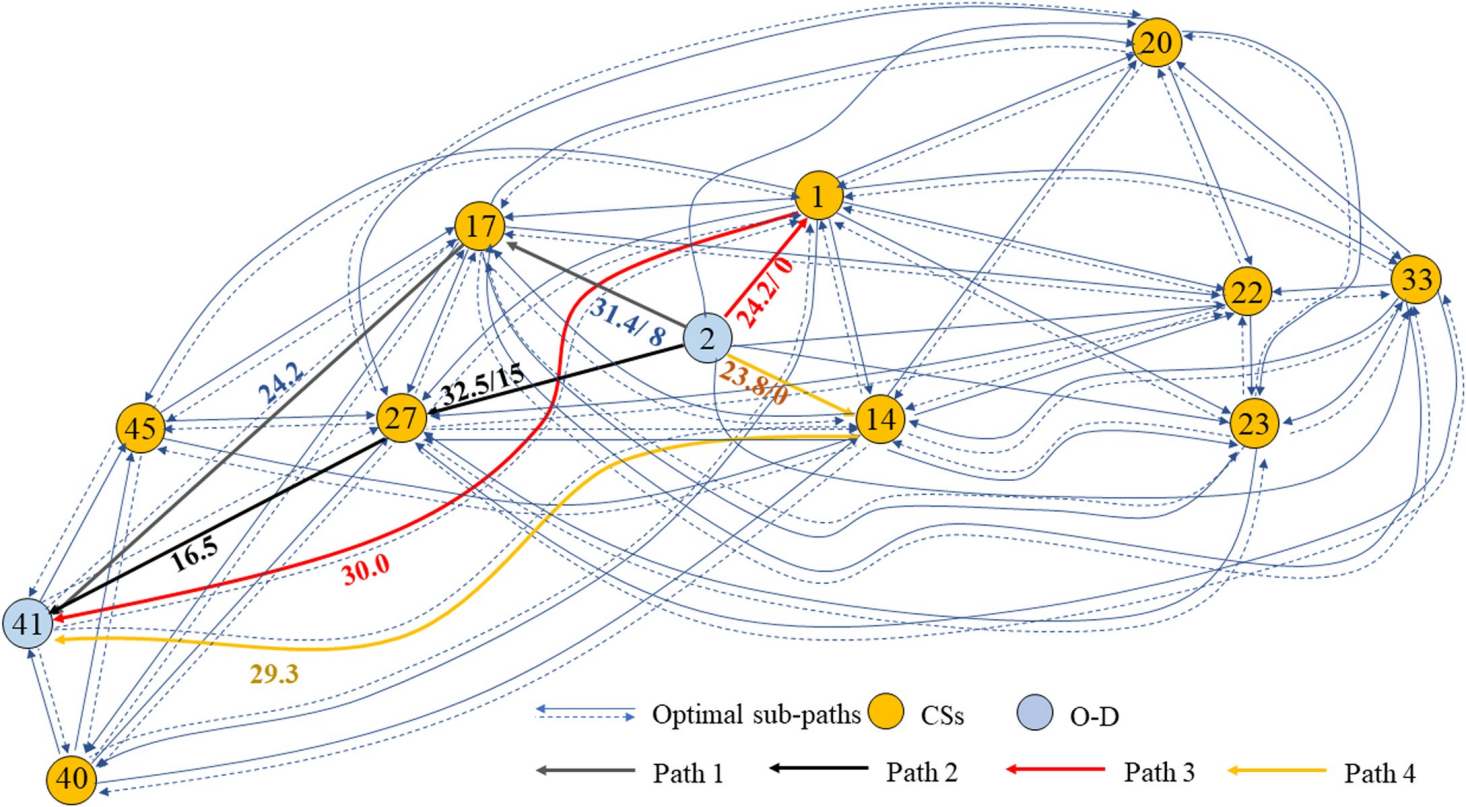
Meta-network and feasible paths of an example EV.

The waiting time at a CS is an important component of the travel cost, especially at peak hours. Ignoring the waiting time may affect the charging decision of EVs. Fig 5 presents the meta-network of the same example EV from Node 2 to Node 41. Four feasible paths with various selected CSs are represented by green, black, red and yellow lines. The basic travel cost (the travel cost regardless of queueing at a CS, namely, a sum of the travel time, the base time loss and the charging time) and the waiting time are plotted in the same color as the corresponding path.

The values of basic travel costs are 55.6 min, 49.0 min, 54.2 min, and 53.1 min, as presented in Table 5. Path 2 has the minimum basic travel cost. The travel cost values are 63.6 min, 64.0 min, 54.2 min, and 53.1 min. Path 4 has the minimum travel cost. Thus, the example EV will charge at CS 14 along Path 4 and spend 53.1 min if the waiting times at the CSs are considered; otherwise it will charge at CS 27 along Path 2 and spend 64 min. Prediction of the waiting times at CSs can facilitate the selection of optimal paths by EVs.

**Table 5:** Travel costs of the four feasible paths for the same example EV from Node 2 to Node 41.

Path	Travel cost (min)	Basic travel cost (min)	Waiting time	Selected CS
**1**	63.6	55.6	8	17
**2**	**64.0**	**49.0**	**15**	**27**
**3**	54.2	54.2	0	1
**4**	**53.1**	**53.1**	**0**	**14**

### Performance comparison

This section applies the EV-TOP and the EV-SWP to 400 EVs of three types in the study area and in 20 randomly generated networks to compare their performances.

Table 6 presents important details on each randomly generated network *G*^*g*^, including the number of vertices |*V*^*g*^|, the number of arcs |*E*^*g*^|, the number of CSs |*S*^*g*^|, and the number of each type of EVs (*Q*_1_^*g*^, *Q*_2_^*g*^, or *Q*_3_^*g*^).

**Table 6:** Important details on the 20 randomly generated networks.

*G*^g^	|*V*^*g*^|	|*E*^*g*^|	|*S*^*g*^|	*Q*_1_^*g*^	*Q*_2_^*g*^	*Q*_3_^*g*^
*G*^1^	60	207	18	192	148	60
*G*^2^	58	184	17	164	124	112
*G*^3^	39	132	6	208	140	52
*G*^4^	44	146	6	240	144	16
*G*^5^	62	220	12	240	132	28
*G*^6^	38	135	4	172	140	88
*G*^7^	36	118	6	164	132	104
*G*^8^	43	139	8	188	136	76
*G*^9^	32	120	5	180	148	72
*G*^10^	53	195	12	212	144	44
*G*^11^	63	207	14	168	140	92
*G*^12^	40	160	7	220	136	44
*G*^13^	50	177	7	208	144	48
*G*^14^	55	215	8	224	124	52
*G*^15^	35	107	6	196	152	52
*G*^16^	46	152	7	164	136	100
*G*^17^	30	103	7	164	144	92
*G*^18^	31	100	9	228	128	44
*G*^19^	52	187	12	232	140	28
*G*^20^	57	228	11	208	144	48

#### Comparison of optimal paths

The optimal solutions of the EV-TOP and the EV-SWP are denoted by X^*1^ and X^*2^, respectively. These EVs differ in terms of origins and destinations. Table 7 lists examples and comparisons. Each row presents the details of the optimal paths for each EV, which include the battery capacity, the initial battery level, the origin, the destination, the total travel times of X^*1^ and X^*2^, the total distances of X^*1^ and X^*2^, the average speeds of X^*1^ and X^*2^, the electricity consumptions of X^*1^ and X^*2^, and the numbers of charging operations of X^*1^ and X^*2^.

**Table 7:** Comparison of the optimal paths of the EV-TOP and the EV-SWP.

EV	*B* kw∙h	*b*_0_ kw∙h	*o*	*d*	Total travel time min		Total distance km		Travel speed km/h		Consumption kw∙h		Number of charging	
					X^*1^	X^*2^	X^*1^	X^*2^	X^*1^	X^*2^	X^*1^	X^*2^	X^*1^	X^*2^
**1**	5	2.4	47	34	70.1	94.2	22.10	18.00	18.92	11.47	4.65	5.19	3	3
**2**	5	2.0	28	7	41.1	39.5	9.23	8.28	13.47	12.58	2.29	2.71	1	2
**3**	50	1.7	38	43	21.4	21.4	8.02	8.02	22.49	22.49	0.97	0.97	0	0
**4**	5	3.0	47	32	67.1	108.0	21.32	18.69	19.06	10.38	4.63	5.48	3	4
**5**	50	2.1	37	26	71.5	91.0	8.92	7.57	7.48	4.99	1.49	1.53	1	1
**6**	20	2.6	28	7	65.6	68.0	9.23	8.28	8.44	7.31	2.26	2.27	1	1
**7**	5	3.2	12	37	21.2	21.2	5.91	5.91	16.75	16.75	1.71	1.71	0	0
**8**	5	2.4	32	44	74.5	82.3	21.10	19.97	16.99	14.56	4.5	4.52	2	3
**…**	…	…	…	…	…	…	……	……					…	…
**399**	20	3.6	25	37	19.3	19.3	4.02	4.02	12.54	12.54	1.00	1.00	0	0
**400**	5	2.8	44	4	41.7	41.7	8.32	8.32	11.97	11.97	2.21	2.21	1	1
**5 (192 EVs)**		Average value			52.3	69.1	13.96	12.73	15.68	11.54	3.21	3.59	1.54	1.84
**20 (156 EVs)**		Average value			58.6	60.3	8.46	7.99	10.52	9.98	2.08	2.13	0.87	0.87
**50 (52 EVs)**		Average value			47.7	50.8	7.94	7.17	14.59	13.78	1.15	1.18	0.62	0.62
**Total**		Total value			21774	25459	4440	4088	-	-	1017	1079	466	524

Comparing the values in each pair of columns, EVs travel longer distances but spend less total travel time if they arrive at their destinations along X^*1^ rather than X^*2^. The total distance along X^*1^ increases 8.6%, while the total time savings that is based on the EV-TOP is up to 3685 min, which represents 14.5% of the total travel cost based on the EV-SWP. Furthermore, the smaller average speed and lower electricity consumption along X^*1^ indicate that the EV-TOP trades a small increase in the distance for substantial decreases in the travel time and the electricity consumption. EVs of the first type stop fewer times for charging along X^*1^ compared to EVs of the other two types. That is because the latter two have high battery capacity while the study area is small. EVs can arrive at any node from any node with a full battery. The advantage of reducing the number of charging operations can be more readily observed on a larger road network, for example, a highway network.

**Fig 6:**
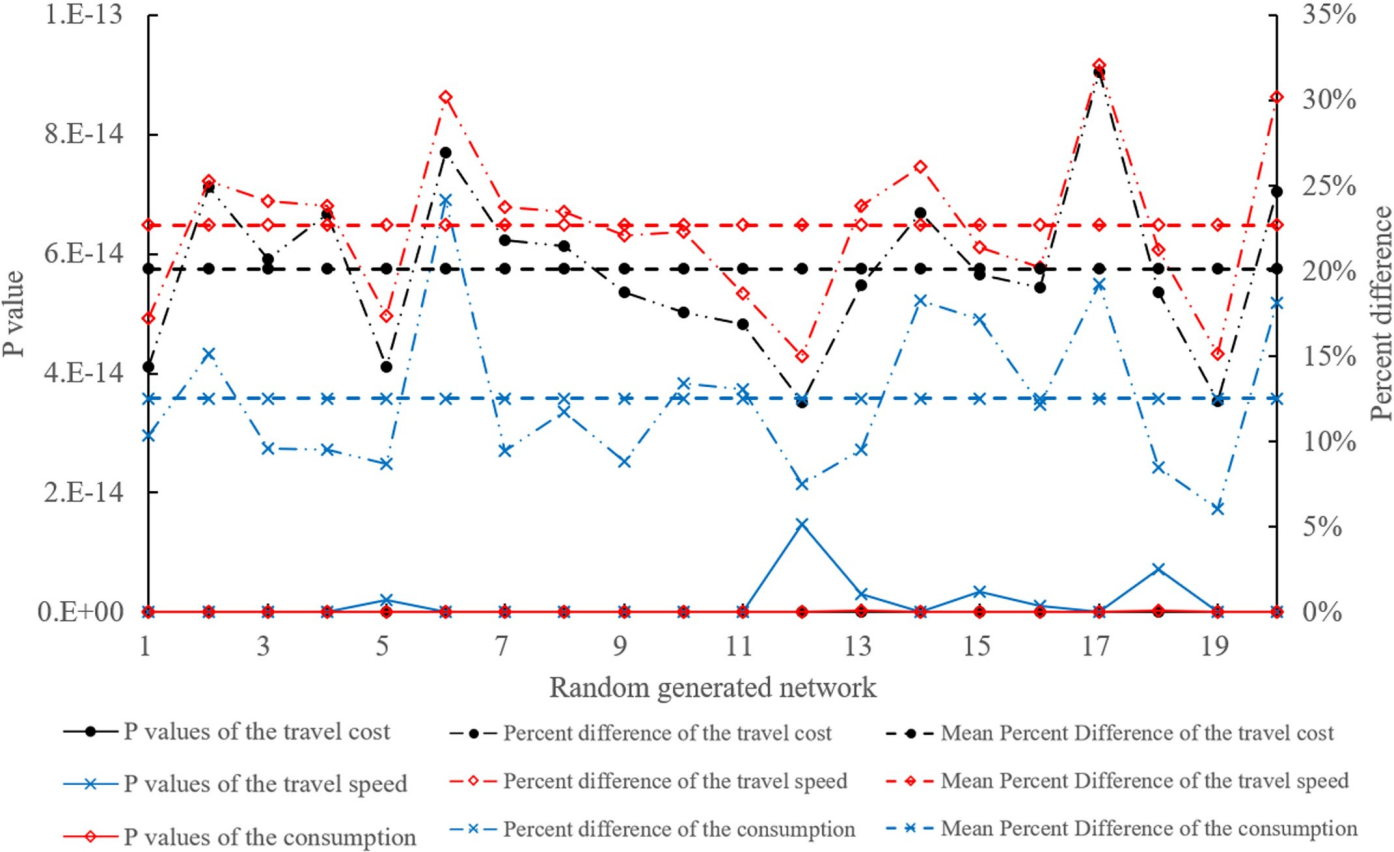
Friedman test results and mean percentage differences of the travel cost, the travel speed and the consumption in the 20 randomly generated networks.

The other two types of EVs charge once at most. The charging operation and optimization method have little influence on the optimal path. To further analyze the significances and the origins of the time savings and the consumption savings, the travel cost, the travel speed, and the consumptions of X^*1^ and X^*2^ for each EV of the first type in the 20 randomly generated networks are considered. The Friedman test is a nonparametric test that compares two or more matched or paired groups. It is conducted to identify significant differences in the travel cost, the travel speed and the consumption between X^*1^ and X^*2^ for each EV. Each randomly generated network *G*^*g*^ corresponds to three Friedman tests that use *Q*_1_^*g*^ paired travel costs, *Q*_1_^*g*^ paired travel speeds, and *Q*_1_^*g*^ paired consumptions. Fig 6 presents the results of the Friedman tests and the mean percentage differences.

The mean percentage differences of the travel cost and the consumption are 20.11% and 12.53%, respectively. All the significance probabilities of the travel cost and the consumption in the 20 randomly generated networks are far less than 0.05. Hence, the improvement of the EV-TOP is significant in terms of time savings and consumption savings. Meanwhile, the travel speed average increases up to 22.68%. Its significance probabilities in the 20 randomly generated networks are far less than 0.05. Therefore, the significant improvements of the EV-TOP in terms of time savings and consumption savings are due to the avoidance of congested links and CSs.

#### Comparison of the LOSs at the CSs

The optimization methods and the corresponding optimal paths of 400 EVs also influence the level of service at each CS. We compare the charging details and the LOSs at ten CSs to further analyze the performance of the EV-TOP. The LOS at a CS depends on the stops for charging, the charging time, and the CS capacity, among other factors. Many criteria could be used. We use the average number of stops for charging per charging pile (*p*_ave_) and the average waiting time per stop (*w*_ave_) to reflect the LOS.

**Fig 7:**
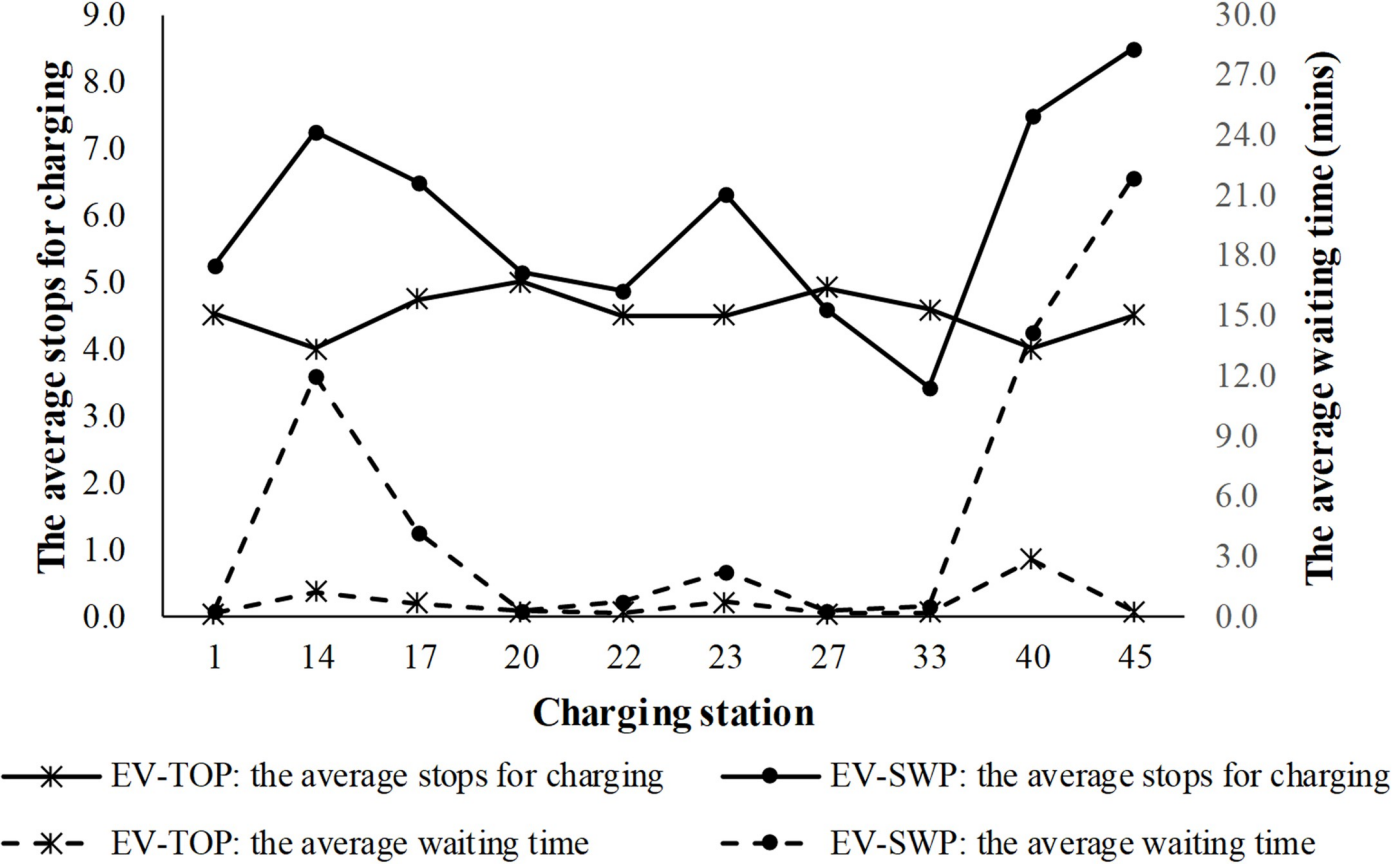
Service levels at each charging station under the two path optimization methods.

Fig 7 plots the average stops for charging per charging pile and the average waiting time per stop at ten CSs based on the EV-SWP and the EV-TOP in different lines. The values at CS 14, CS 23, and CS 45 differ significantly between the two methods. In addition, the average number of stops for charging and the average waiting time based on the EV-TOP are relatively low. They also differ little among the ten CSs. In contrast, those values are much higher and fluctuate substantially with CSs. Hence, EV-TOP can balance the LOSs at CSs by reducing the number of stops for charging and by selecting the more reasonable CSs. Thus, the waiting time at CSs declines and the LOS improves. This is further supported by the Friedman test, which is conducted later.

The number of stops for charging, the base time losses, the waiting times, and the charging times at ten CSs for the EV-TOP and the EV-SWP are listed in Table 8. Comparing each pair of rows, in addition to the total number of stops for charging, the total base time loss, the total waiting time, and the total charging time are reduced by the EV-TOP. The time savings are 72 min, 2239 min, and 291 min, respectively, for a total of 2602 min. They account for 70.6% (2602/3685*100%) of the total time savings. Hence, the improvement of the EV-TOP relies mainly on the decrease of the waiting time at CSs, followed by the decrease of the travel time and the decrease of the charging time.

**Table 8:** Charging details at each charging station under the two path optimization methods.

Measures	Method	CS										Total	Difference
		1	14	17	20	22	23	27	33	40	45		
**Stops for charging**	EV-TOP	95	16	19	60	36	27	59	110	8	36	466	58
	EV-SWP	110	29	26	62	39	38	55	82	15	68	524	
**Base time loss** **min**	EV-TOP	456	35	55	114	40	70	148	561	16	61	1556	72
	EV-SWP	528	64	75	118	43	99	138	418	30	116	1628	
**Waiting time** **min**	EV-TOP	14	20	13	17	8	19	9	21	23	9	153	2239
	EV-SWP	34	347	110	19	31	86	20	44	212	1489	2391	
**Charging time** **min**	EV-TOP	2044	344	409	1291	774	581	1269	2366	172	774	10024	291
	EV-SWP	2165	571	512	1220	768	748	1083	1614	295	1339	10315	

Similarly, the Friedman tests are conducted to identify significant differences in the average number of stops for charging per charging pile and the average waiting time per stop among the ten CSs in the 20 randomly generated networks. The results of the four Friedman tests on the 20 groups of ten-matched *p*_ave_ based on the EV-TOP, the 20 groups of ten-matched *p*_ave_ based on the EV-SWP, the 20 groups of ten-matched *w*_ave_ based on the EV-TOP, and the 20 groups of ten-matched *w*_ave_ based on the EV-SWP are presented in Table 9. The test results demonstrate that the EV-SWP results in significantly different average numbers of stops for charging and average waiting times among the ten CSs. In contrast, there is no significant difference in the average number of stops for charging or the average waiting time using the EV-TOP. Thus, the EV-TOP can balance the LOSs at CSs.

**Table 9:** Friedman tests results for the average number of stops for charging and the average waiting time among CSs.

	Average stops for charging		Average waiting time	
**Method**	EV-TOP	EV-SWP	EV-TOP	EV-SWP
**P value**	0.334	0.000	0.430	0.000

According to the comparisons of the optimal paths and the LOS at each CS, the performance of the EV-TOP is improved by 1) trading a small distance increase for a significant travel time decrease and 2) balancing the LOS at each CS.

## Conclusions

The EV-TOP that is proposed in the paper aims at identifying the optimal path for EVs by minimizing their travel cost, which is composed of the travel time and the total time that is spent at CSs and is limited by the battery level. The main contributions of this paper are as follows:

1. More accurate approximations of the electricity consumption, the waiting times at CSs, and the charging function improve the accuracy of the travel cost. The optimal path will be much more reasonable and convincing.
2. The optimization considers the traffic state, the LOSs at CSs, the reliability preferences of the EV, and the charging operations. A small distance increase is traded for a substantial travel time savings. Thus, comparing with other methods, the EV-TOP will not produce a shorter but much more congested path. EVs can reduce their numbers of stops for charging and spend less time at CSs. The LOS at each CS can also be balanced.
3. The EV-TOP is decomposed into two subproblems: a constrained optimal path problem in the actual network (EV1-COP) and a specified shortest path problem in the meta-network (EV2-SP). The solution process is simplified. A polynomial-time approximation algorithm is proposed for the EV-TOP. However, EVs of the same type during the same period can share the same meta-network, except for the subpaths from the origin *o* to the destination *d*. This will improve the efficiency of the system for all the EVs.

As discussed above, the EV-TOP may perform better on a highway network. In the future, the difference between applying the EV-TOP in an urban network and in a highway network will be analyzed. The EV-TOP will be further developed. In addition, the improvement of the utility function and the determination of various parameters, such as the optimal travel speed, are outside the scope of the paper. In the future, their optimal values will be approximated using a much larger amount of real data and their impacts on the solution will be analyzed.
